# Who will pass? Analyzing learner behaviors in MOOCs

**DOI:** 10.1186/s41039-016-0033-5

**Published:** 2016-04-08

**Authors:** Shu-Fen Tseng, Yen-Wei Tsao, Liang-Chih Yu, Chien-Lung Chan, K. Robert Lai

**Affiliations:** 1 Department of Information Management and Innovation Center for Big Data and Digital Convergence, Yuan Ze University, 135 Yuan-Tong Road, Chung-Li District Tao-Yuan City, Taiwan; 2 Department of Information Management, Yuan Ze University, 135 Yuan-Tong Road, Chung-Li District Tao-Yuan City, Taiwan; 3 Department of Computer Science and Engineering and Innovation Center for Big Data and Digital Convergence, Yuan Ze University, 135 Yuan-Tong Road, Chung-Li District Tao-Yuan City, Taiwan

**Keywords:** MOOCs, Learning engagement, Learning behavior, Learning analytics

## Abstract

Massive open online courses (MOOCs) have recently gained worldwide attention from educational institutes. MOOCs provide a new option for learning, yet measurable learning benefits of MOOCs still need to be investigated. Collecting data of three MOOCs at Yuan Ze University (YZU), this paper intended to classify learning behaviors among 1489 students on the MOOC platform at YZU. This study further examined learning outcomes in MOOCs by different types of learners. The Ward’s hierarchical and k-means non-hierarchical clustering methods were employed to classify types of learners’ behavior while they engaged in learning activities on the MOOC platform. Three types of MOOC learners were classified—active learner, passive learner, and bystander. Active learners who submitted assignments on time and frequently watched lecture videos showed a higher completion rate and a better grade in the course. MOOC learners who participated in online discussion forum reported a higher rate of passing the course and a better score than those inactive classmates. The finding of this study suggested that the first 2 weeks was a critical point of time to retain students in MOOCs. MOOC instructors need to carefully design course and detect risk behaviors of students in early of the classes to prevent students from dropping out of the course. The feature design of discussion forum is to provide peer interaction and facilitate online learning. Our results suggested that timely feedback by instructors or facilitators on discussion forum could enhance students’ engagement in MOOCs.

## Background

The development of massive open online courses (MOOCs) has been recognized as one of the disruptive innovations in the field of education (Jacoby 2014). A MOOC is “an online course with the option of free and open registration, a publicly shared curriculum, and open-ended outcomes” McAulay et al. (2010). In recent years, there has been considerable debate over the MOOC phenomenon in academic cycles. MOOC advocates claimed the open nature of MOOCs could offer some unique opportunities for pedagogical change and provide transformative effects on both teaching and learning. Yet the pedagogical approach of MOOCs is under scrutiny since some research suggests that large-scale lectures and demonstrations do not necessarily give rise to student learning (Daniel 2012). Several issues are commonly being discussed, such as the impact of MOOCs on university teaching practice, types of MOOCs, quality assurance and accreditation, definition of success, and course completion (Blake 2014; Clow 2013; Hew and Cheung 2014; Jacoby 2014; Margaryan et al. 2014; Schulze 2014).

While a general recognition of the increase in MOOCs is found, evidence about the pedagogy of learning in MOOCs remains limited Mackness et al. (2013). In MOOCs, a collection of video lectures, readings, quizzes, peer-graded assessments, and discussion forums is featured to pull learners together. Glance et al. (2013) conducted a narrative analysis to explore whether MOOCs represent a pedagogically sound format for higher education. Their findings suggested that MOOCs are no less an effective learning experience than those of traditional practices. They claimed that online quizzes and assessments could enhance learning through the mechanism of retrieval practice, short videos allow for mastery learning and complement the optimal attention span of students, and discussion forums help to provide peer assistance interaction.

Despite the high degree of interest and the rapid development of MOOCs, we still lack enough understanding about how students engage in such courses (Anderson et al. 2014). The high dropout rate on most MOOCs is the biggest challenge faced by online education providers. MOOC features of short videos, online quizzes and assessments, peer assessment, and discussion forum are designed to motivate and enhance students’ learning. Yet whether MOOCs result in a better learning outcome is now needed to be explored in-depth. Recent research has suggested that quantitative and qualitative studies are needed to investigate the academic potential and measurable learning benefits of MOOCs (Boyd and Kasraie 2013).

After the first American MOOCs launched by Stanford University in 2011, many of the world’s elite universities are now offering some of their best courses for people to learn free online. In Taiwan, Yuan Ze University (YZU) is one of the few universities that provide MOOCs. The university has created its own MOOC platform and provided five MOOCs to students in 2014. We collaborated with the Office of Information Services at campus and collected learners’ footprints and their learning patterns from MOOCs at YZU. By analyzing learners’ behaviors in MOOCs and their impacts on learning outcome, the main objective of this study is to understand how students engage in MOOCs and offer insight to what engages them in MOOC environments. Students’ performance in MOOCs was examined to expand our knowledge about how students respond to these pedagogical practices.

## Literature review

MOOCs have created great opportunities for educators and researchers who are interested in learning analytics. The huge number of students who participate in MOOCs means that educators have access to large data sets of students’ online learning interactions. Through the techniques of learning analytics, large sets of educational data can be used to develop a greater understanding of students’ online behaviors, engagement patterns, and their learning outcome (Coffrin et al. 2014). A review of studies on learner engagement, types of MOOC learners, and learners’ performance in MOOCs were presented as follows.

### Learner engagement and types of MOOC learners

Kizilcec et al. (2013) analyzed patterns of engagement and disengagement in three MOOCs on the Coursera platform. Students were classified based on their engaging behaviors of watching video lectures and assessments submission. They labeled four types of learner engagement: “on track” (did assignment on time), “behind” (delayed assignment), “auditing” (did not do assignment but watched videos or took tests), and “out” (did not participate in course at all). They further employed cluster analysis and classified students into “completing,” “auditing,” “disengaging,” and “sampling” patterns of engagement. Completing pattern of engagement characterized learners who completed majority of assessments. Auditing learners were those who watched most of the videos but completed assessments infrequently. Disengaging learners completed assessments at the start of the course but withdrew engagement afterwards. Sampling learners merely explored some course videos. Anderson et al. (2014) also used two activities: viewing a lecture video and handing in an assignment for credit in six MOOCs on Coursera to distinguish five styles of learning engagement. They defined five patterns of students’ engagement: the viewers, solvers, all-rounders, collectors, and bystanders. Viewers primarily watched course lectures, solvers primarily handed in assignments for a grade, all-rounders suited in the middle of watching lectures and handing in assignment. Collectors downloaded lectures but may not be actually watching them. Bystanders registered for the course, but their total activity was below a very low threshold. In their study, all these clusters were found consistently across MOOCs that were designed for students at a different educational level.

Recognizing the importance of learners’ intention on learning, Koller et al. (2013) distinguished MOOC learners into “browsers” and “committed learners.” They further differentiated committed learners into passive participants, active participants, and community contributors. Passive learners were those who frequently watched lecture videos, but have limited participation on course forums, and attempted few assignments and quizzes. Active learners enthusiastically engaged in the course by completing homework assignments, quizzes, exams, and peer-graded assessments. Community contributors also actively participated in courses, but they showed specific interests on facilitating forum discussion and making contributions to the public good.

Ferguson and Clow (2015) took the social constructivist approach and claimed that contributing to or reading discussion comments was an important part of the learning process in MOOCs. They analyzed four FutureLearn MOOCs and found that only completing and sampling clusters identified previously by Kizilcec and his colleagues (2013) can be applied in their cases. Yet, when active contribution to discussion was included in cluster analysis, seven distinct clusters were emerged. Two most successful clusters appeared to be learners who extensive and structured use of discussion comments. In this approach, knowledge is jointly constructed through conversation.

### Student engagement and performance

Anderson et al. (2014) investigated how a student’s level of engagement and activity correlated with his/her final grade. They found that the main characteristic of high achievers was that they consumed many lecture videos. Findings of two studies (Karpicke and Roediger 2008; Karpicke and Blunt 2011) also showed that online learners who watched videos did have a better learning performance. In particular, reviewing short quizzes in the videos could improve students’ learning outcome. Santos et al. (2014) analyzed students’ behaviors in MOOCs and found that students who participated more on courses activities have a better chance to pass the course. Those students who frequently communicated, discussed, shared, and collaborated with others showed a better learning outcome. Their study also suggested that those who posted often in discussion forum would have a higher rate of passing the course.

## Methods

YZU is one of the few universities in Taiwan offers MOOCs. Five MOOCs were launched at YZU in 2014. They were “C# Programming” (400 enrolled students), “Internationalization Strategy” (340 students), “Computer-aided Design and Manufacture” (749 students), “Electronics: Amplifier Principles and Analysis” (193 students), and “English for Engineering & Technology” (381 students). Since only the first three courses provided final grades to the MOOC system, students’ behavioral data from logging in system, watching lecture videos, submitting assignments, and posting on discussion forums were collected from these three courses to examine engagement patterns of MOOC students. Totally, we collected data from 1489 students, with a gender distribution of 54 % male students and 46 % female students. Among these students, about 63 % of students fell into the 16 to 25 years old category, 18 % into the 26–35 age group, 12 % into the 36–45 age group, and 7 % of students were over 45 years of age. There were only 6 % of non-Taiwanese registered students in these MOOCs.

Cluster analysis can be used to help researchers develop profiles that are grounded in learner activities (Antonenko et al. 2012). This study used the Ward’s hierarchical and k-means non-hierarchical clustering methods to determine the number of clusters and to classify different clusters of learners in MOOCs. The Ward’s minimum variance clustering is useful for exploratory work when researchers do not have a preconceived number of clusters in the dataset. It uses an analysis of variance approach to evaluate the distances between clusters. Ward’s algorithm compares the proximity indices and identifies pairs of participants with the smallest distance value. Once we identified the number of clusters, k-means clustering method was used to analyze learning behaviors in different clusters of MOOC students. The k-means clustering method calculates centroids for a set of trial clusters and then places each object in the cluster with the nearest centroid; this process continues until there are no more changes in the cluster membership (Antonenko et al. 2012).

Descriptive analyses, including chi-square and mean-difference tests were conducted to compare students’ learning outcomes in different clusters. There were two indicators for learning outcome: whether students passed the courses and their final grades of MOOC course. In addition, different levels of participating in course discussion forum were examined to explore its impacts on learning by descriptive analyses.

## Results

### Learning engagement in MOOCs

**Fig. 1:**
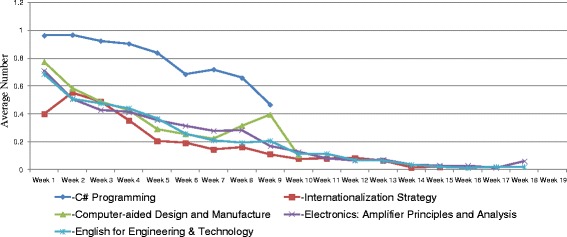
The average number of logging in MOOCs

**Fig. 2:**
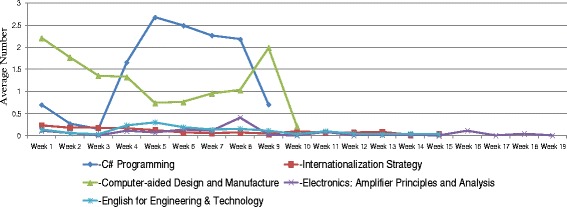
The average number of watching lecture videos

**Fig. 3:**
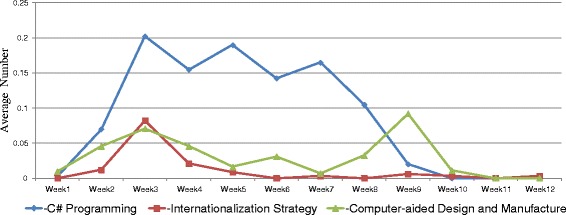
The average number of assignment submission

Trends of students’ learning behaviors in MOOCs, including login records, lecture video watching, and assignment submission were analyzed. Figure 1 shows the average numbers of logging in five MOOCs. In general, the average numbers of logging in these courses were relatively low. Only students in the “C# Programming” course have closely reached the average number of 1 in the first 2 weeks, indicated that students in this class logged in the system once in a week during the first 2 weeks of class. After the second week, the average numbers of logging decreased over the course weeks. Frequently watched lecture videos were merely seen in the “C# Programming” and “Computer-aided Design and Manufacture” courses (Fig. 2). The highest numbers of video watching in the “C# Programming” course were the weeks that assignments were worth large proportion of their final grades. The number of video watching was peaked at the ninth week of “Computer-aided Design and Manufacture” course mainly because of the impact of invited speaker on the lecture. Students in the other three courses showed an inactive use of lecture videos. Figure 3 shows that students in the “C# Programming” course also revealed a higher average number of submitting assignments over the whole 9-week course than the other courses.

### Classify learners by cluster analysis

We collected students’ behavioral data from three courses (“C# Programming,” “Internationalization Strategy,” and “Computer-aided Design and Manufacture”) to identify types of learners in MOOCs. A cluster analysis was performed to examine learning profiles based on students’ engagement in logging into the portal, watching course videos, and handing in assignments. The cluster analysis was used to identify homogeneous groups of cases, in which the cases within a cluster shared similarity but were dissimilar to the cases included in other clusters. For each of the course, an agglomerative hierarchical clustering method (Ward’s method) followed by an iterative, non-hierarchical (k-means) clustering technique was used. The Ward’s clustering results of these courses suggested a three-cluster solution as best fitting the data.

Once the number of clusters in each of the MOOC was decided by the Ward’s method, the k-means clustering analysis was employed. K-means clustering method helps to classify students and describe students’ engagement in each of the cluster. Among 1489 total students in three courses, 18 of them were classified in the first cluster, 127 students were in the second, and 1344 in the third cluster, respectively. In the “C# Programming” course, there were 7 students classified into the first cluster, 52 students in the second, and 341 students in the third cluster. In the “Computer-aided Design and Manufacture” course, there were 11 students grouped into the first cluster, 65 students in the second, and 673 students in the third cluster. We followed the classification of previous studies (Koller et al. 2013; Kizilcec et al. 2013) and named three types of learners: active learner, passive learner, and bystander. In the “C# Programming” course, the average number of watching lecture videos during the course period was 155 times for active learners, compared to 63 times for passive learners, and only 2 times for bystanders. Active learners in the “Computer-aided Design and Manufacutre” course showed an average of 198 times in watching course videos, compared to 68 times among passive learners and 5 times among bystanders. Completing homework assignments were more often among active learners than passive learners and bystanders. In all three courses, only few of bystanders had ever handed in course assignments.

Table 1 shows the numbers and proportions of different types of learners. In three MOOCs, most students were classified as bystanders (90 %), only 1 % of students were active learner, and 9 % of them were passive learners. While the “C# Programming” course had the highest proportions of active (2 %) and passive (13 %) learners, students in the “Internationalization Strategy” course composed most of bystanders (97 %). Consistent with global trends of MOOC learning, course completion rates in MOOCs were relatively low, ranged from 5–15 % of initial enrollees (Glance et al. 2013). In this study, there were only 97 out of 1489 students (6.5 %) who passed their courses in MOOCs.

**Table 1:** Numbers and proportions of different types of learners in MOOCs

Courses	Active learner	Passive learner	Bystander
C# Programming	7 (2 %)	52 (13 %)	341 (85 %)
Internationalization Strategy	0 (0 %)	10 (3 %)	330 (97 %)
Computer-aided Design and Manufacture	11 (1 %)	65 (9 %)	673 (90 %)
All courses	18 (1 %)	127 (9 %)	1344 (90 %)

### Learning performance in three clusters

Chi-square and mean-difference (one-way ANOVA) tests were conducted to measure learning performance in different types of learners (Table 2). The results indicated that students with various types of learning behaviors in MOOCs did reveal different levels of learning outcome. Active learners who completed homework assignments and watched videos frequently have significantly shown higher rates of passing the courses than the others. In all, while 42 % of active learners passed the courses, only 33 % of passive learners and 3 % of bystanders completed their MOOCs.

**Table 2:** Course completion rates and score by different types of learners in MOOCs

Courses	Completion rate	Average score	Significance
C# Programming	Total = 16 %		
Active learner	85 %	84	***
Passive learner	63 %	68	
Bystander	8 %	9	
Internationalization Strategy	Total = 5.6 %		
Active learner	–	–	
Passive learner	20 %	68	n.s.
Bystander	5 %	48	
Computer-aided Design and Manufacture	Total = 1.7 %		
Active learner	27 %	54	***
Passive learner	14 %	43	
Bystander	0 %	15	
All courses	Total = 6.5 %		
Active learner	42 %		
Passive learner	33 %		
Bystander	3 %		

***p < 0.001

*n.s.* not significant

The course completion rate was highest in the “C# Programming” course. The overall passing rate of this course was 16 %. There was a proportion of 85 % active learners in the “C# Programming” course completed the course, compared to that of 63 % for passive learners, and less than 8 % for bystanders. The average grades of “C# Programming” course were 84 for active learners, 68 for passive learners, and 9 for bystanders. Both the results of chi-square and mean-difference tests suggested that learning outcomes were significantly different across active, passive, and bystander groups (*p* < .001). Instructor in this class reported that short quiz reviews after a session, peer assessment on assignments and timely feedback on discussion forum enhanced students’ engagement in this class.

In the “Internationalization Strategy” course, the pass rate was low at 5.6 % of total enrolled students. There were two clusters of students identified in this course: passive learners and bystanders. No active engagement learner was clustered in this course which might due to the course was designed for proficient English speakers, thus hindered non-native English speaking students from understanding learning materials. While 20 % of passive learners passed the course, only 5 % of bystanders eventually completed the course. The learning outcomes of these two groups were not significantly different by chi-square and mean-difference tests.

The pass rate for “Computer-aided Design and Manufacture” course was relatively low than the other two courses. Only 13 students out of total 749 enrolled students (1.7 %) completed the course. The below average completion rate in this course might due to a final work exhibition requirement of this class. In this course, there were 3 out of 11 active learners (27 %), 9 out of 65 passive learners (14 %), and 1 out of 673 bystanders passed the course. The average scores of active, passive, and bystander groups in this course were 54, 43, and 15, respectively. The chi-square and mean-difference tests suggested that the proportions of course completion and final grades were significantly different across active, passive, and bystander groups (*p* < . 001).

### Impact of discussion forum

In this section, we examined the impact of discussion forum on course completion rates and final scores. Only the “C# Programming” course applied this feature on course design at the YZU MOOC platform. A discussion forum was provided for students to discuss and collaborate with each other while they undertake their learning in the course. Those who had ever posted or responded in discussion forum of this course were categorized as forum users, and those who never participated in the discussion forum were classified as inactive users. Chi-square and mean-difference tests were employed to explore the effects of forum usage in MOOCs. A total of 8 % students in this class participated in the class forum. The pass rates of forum users and inactive users and their average grades of this course were calculated. The results indicated that the pass rate for forum users was 68 % compared to only 11 % of inactive users who eventually completed the course. Chi-square test showed a significant difference (*p* < .001) between forum users and inactive users, with a chi-square value at 72.1. The test of mean difference also revealed a better grade of forum users (mean score of 72) in compared to that of inactive users (mean score of 13) in their final scores (Table 3).

**Table 3:** Course completion rates by discussion forum users in the C# Programming course

C# Programming	Completed	Incomplete	Significance
Forum users	23 (68 %)	11 (32 %)	***
Average score = 72			
Forum inactive users	42 (11 %)	324 (89 %)	
Average score = 13			

***p < 0.001

## Discussion and conclusion

In this study, we first described patterns of learning engagement in three MOOCs. Then, the Ward’s and k-means cluster analyses were employed to classify students by their learning behaviors. Further, we examined the relationship of different clusters of MOOC learners and their learning performance. Same as previous MOOC studies have shown (Cassidy et al. 2014), the completion numbers of MOOC learners in this study were underwhelming. The trends of logging in records in this study suggested that learners withdrew as early as in the second week of the courses. Except for two courses, enrolled students showed a low frequency of logging in system and few number of watching lecture videos. Records of learning engagement among these students indicated that the first 2 weeks was a critical point of time to retain students in MOOCs. For students’ retention, MOOC instructors need to carefully design course sections and pay more attention to students’ feedbacks in early of the classes. Research by Ferguson and Clow (2015) found that there were several points at which learners may leave a course. They suggested that tight linkage between weekly learning materials and pointing learners forward might reduce the drop-off, particularly at the ends of week 1 and week 2. Secondly, learners in MOOCs at YZU can be classified into three groups: active learner, passive learner, and bystander. The results of cluster analyses showed that only 1 % of students were active learners, while the rest of 9 and 90 % of students were clustered as passive learners and bystanders. Active learners did show a higher completion rate and a better final grade than the other groups. These results suggested that learning performances of MOOC students varied by their learning engagement and participation in learning activities. The participation of discussion forum in MOOCs did result in better outcomes suggested that this feature design in MOOCs could provide peer interaction and enhance online learning.

Understanding how students interact with MOOCs is crucial, because it affects how we design MOOC courses and how we evaluate their effectiveness (Anderson et al. 2014). The findings of this research contribute to a better understanding of learners’ engagement and performance in MOOCs. The patterns of learning engagement of active and passive learners in this study were similar to the clusters of completing and sampling learners in the study conducted by Kizilcec and his colleagues (2013). Research on different types of learning engagement can help inform a range of strategies for intervention and improvement in MOOCs. In this study, a small proportion of students were clustered as active learners who watched lecture videos frequently and handed in assignments for credit. Whether the better outcomes of this group resulted from personal positive attitude toward MOOC learning or from specific pedagogical design that enhanced learning performance needs to be further explored. Compared to active learners, passive learners watched about one third of lecture videos and handed in one half of assignments. The intermittent participation of passive learners in MOOCs might relate to course workload, task designs, and level of facilitation in the class (Cassidy et al. 2014). Further work is required to verify factors that shape learning behaviors of this group of learners. Ferguson and Clow (2015) suggested that providing previews of course material would allow these sampling learners to make a more informed decision about whether to register in the first place. Our findings found that most MOOC learners fell into the “bystander” category. The course completion rate was merely 3 % in this group. Keeping this group of students motivated and engaged in MOOCs is a great challenge. Further research should focus on departure behaviors in the first few weeks among bystanders, profiling their engagement patterns and identifying who are at risk of dropping out of courses.

This study recognized the importance of discussion forum feature on engaging students’ learning. The presence of forum discussion was correlated with higher course retention and students’ performance. A recent study of Sharif and Magrill (2015) has suggested that discussion forums in MOOCs represent a unique opportunity for insight into the formation of learning communities. Active participation in discussion forum might be a key component of successful online courses, yet it requires instructors to review and respond upon student comments, raise questions, and make observations to guide discussions in a desired direction. The scale of MOOCs and the diversity of learners’ experiences make it a challenge for instructors to provide a level of online facilitation that is capable of enhancing students’ learning experience (Cassidy et al. 2014).

Analyzing learners’ behaviors and patterns of engagement help to suggest research and design directions for future courses. Two engagement patterns emerged from this study: active learners completed the course thoroughly and passive learners visited the course periodically. The engaging behaviors and patterns of these two clusters are likely to prove the richest for improving the quality of learning and the learning environment. Further research can interview those in different clusters and profile distinct patterns of engagement to explore factors that leverage learning outcomes among various types of learners. One MOOC in this study (C# Programming) reported an extraordinary retention and completion rates. A further in-depth analysis of the pedagogical activities of teachers for successful MOOCs could provide insight into the best practices for course design in MOOCs. While this study explored students’ engagement and pedagogical design aspects of MOOCs, previous research highlighted the significance of learner’s intrinsic motivation and personal circumstances in their ultimate success. Future work could explore personal perception, attitudes, and cognitive strategies of MOOC learning in different clusters to understand their relationship with learning engagement and attrition.
